# Supplementary material for: Content-rich biological network constructed by mining PubMed abstracts
Source: BMC Bioinformatics. 2004 Oct 8;5:147. doi: 10.1186/1471-2105-5-147 (PMC528731; doi:10.1186/1471-2105-5-147)
Supplement: Additional File 2 — The original results of the above study (non-essential files are deleted to keep the file size under the limit set by BMC bioinformatics). [file 1471-2105-5-147-S2.bz2 › chilibotAdditionalFile2/dip05/532ID9671457E10849/html/NR3C1_HMGB1.html]

 


 **NR3C1** and **HMGB1** 
  
Found 6 abstracts in PubMed, retrieved 05.  
 

 What does Google say? 
 PDF only 
| .edu only 

---

**Interactive relationship** (e.g. stimulation, inhibition, etc)

**Inhibitory relationship**- Cell lines lacking Hmg1  [ **HMGB1** ]  grow normally, but the activation of gene expression by the glucocorticoid receptor  [ **NR3C1** ]  GR, encoded by the gene Grl1 is impaired.  Ref: 10391216 Nat Genet, 1999
**Neutral relationship**

**Non-interactive relationship** (e.g. studied together, co-existance, homology, etc.)

- Using both stable and transient transfection assays, we demonstrate that the overexpression of high mobility group protein I C HMGI C, a Ras ERK inducible HMG  [ **HMGB1** ]  I family member, represses glucocorticoid receptor  [ **NR3C1** ]  GR dexamethasone Dex stimulated alpha ENaC reporter activity in salivary epithelial cells.  Ref: 11390395 J Biol Chem, 2001
